# Supplementary material for: Common inflammatory proteins linking frailty and area-level deprivation as key drivers of cardiovascular risk in women
Source: Commun Med (Lond). 2025 Jul 20;5:301. doi: 10.1038/s43856-025-01012-4 (PMC12276345; doi:10.1038/s43856-025-01012-4)
Supplement: Supplementary file 2 — Supplementary Information [file 43856_2025_1012_MOESM2_ESM.pdf]

Supplementary Information

Supplementary Table 1. Demographic characteristics of the most deprived frail and the least deprived non-frail individuals from TwinsUK

Phenotype	Overall	Most deprived (1st) frail	Least deprived (5th) non-frail	P value
N	2,144	102	151	-
Females, N (%)	2,144(100%)	102(100%)	151(100%)	-
Age, yrs	60.14(8.80)	62.15(9.94)	57.50(8.51)	0.0002
BMI, kg/m2	26.44(4.69)	29.37(6.80)	24.67(3.35)	$2.79x10^{-9}$
IMD	7(4)	3(2.75)	10(0)	$<2.2x10^{-16}$
Frailty index	0.13(0.10)	0.36(0.09)	0.05(0.02)	$<2.2x10^{-16}$
Age stopping full-time education, yrs	17.17(3.07)	16.44(3.62)	17.64(2.55)	$1.94x10^{-9}$
Physical activity, N (%)				
IPAQ Score - Low	515(24.02%)	31(30.39%)	26(17.22%)	0.0003
IPAQ Score - Moderate	1,089(50.79%)	59(57.84%)	76(50.33%)	
IPAQ Score - High	540(25.19%)	12(11.76%)	49(32.45%)	
Healthy Eating Index (HEI)	61.22(9.09)	59.35(9.20)	62.45(8.36)	0.0005
Current smoker, N (%)	190(8.86%)	19(18.63%)	8(5.30%)	0.002
Drinking, N (%)				
Never	332(15.49v%)	17(16.67%)	19(12.58%)	0.136
Social occasions only	944(44.03%)	53(51.96%)	66(43.71%)	
Weekly	868 (40.49%)	32(31.37%)	66(43.71%)	
Blood pressure lowering medications	39(15.41%)	28(27.45%)	11(7.28%)	$2.91x10^{-5}$
Antidiabetic medications	5(1.97%)	5(4.90%)	0(0.00%)	0.022
Antilipemic drugs	51(20.16%)	41(40.20%)	10(6.62%)	$1.89x10^{-10}$

The number in each cell denotes the mean (standard deviation, SD) or median (Interquartile range, IQR) for the continuous variables or count (percentages) for the categorical variables. The Wilcoxon rank-sum test was used to compare the statistical difference in continuous variables. Pearson's chi-squared test was used to identify the difference in categorical variables between groups.

Supplementary Table 2. The association of IMD and frailty related protein markers with ASCVD score in TwinsUK. The beta coefficients were calculated by the linear mixed models adjusting for age, BMI, batch and family relatedness.

**Protein**	**Beta**	**95% CI**	**P value**
IL18R1	0.046	[0.024, 0.067]	3.21×10^{-5}
FGF21	0.059	[0.038, 0.080]	2.20×10^{-8}
CCL11	0.03	[0.009, 0.050]	4.43×10^{-3}
TGFalpha	0.052	[0.033, 0.071]	9.73×10^{-8}
VEGFA	0.053	[0.033, 0.073]	2.39×10^{-7}
TNFSF14	0.043	[0.023, 0.063]	3.35×10^{-5}
FGF19	0.008	[-0.012, 0.027]	4.50×10^{-1}
HGF	0.079	[0.058, 0.100]	1.34×10^{-13}
OSM	0.062	[0.042, 0.082]	8.31×10^{-10}
CDCP1	0.055	[0.033, 0.077]	1.34×10^{-6}

Supplementary Table 3. The association of IMD and frailty related protein markers with ASCVD score in the Nottingham OA cohort. The beta coefficients were calculated by the linear models adjusting for age and BMI.

**Protein**	**Beta**	**95% CI**	**P value**
IL18R1	0.13881	[-0.001, 0.279]	0.052
FGF21	0.09508	[-0.044, 0.234]	0.176
CCL11	0.17388	[0.043, 0.305]	0.010
TGFalpha	0.08482	[-0.049, 0.219]	0.210
VEGFA	0.07875	[-0.055, 0.212]	0.241
TNFSF14	0.18758	[0.060, 0.315]	0.005
HGF	0.13676	[0.005, 0.269]	0.043
OSM	0.05116	[-0.088, 0.19]	0.464
CDCP1	0.19939	[0.070, 0.329]	0.003

Supplementary Table 4. Association between externally validated inflammation-related markers and risk of incident ischemic heart disease in TwinsUK. The hazard ratio (HR) was calculated by the mixed-effect Cox regression models, adjusting for BMI, with batch and family relatedness as random effects in age-matched cases and controls.

**Protein**	**HR**	**95% CI**	**P value**
HGF	0.934	[0.505, 1.729]	0.828
CDCP1	1.824	[1.169, 2.845]	0.008
TNFSF14	1.137	[0.747, 1.73]	0.550
CCL11	1.048	[0.583, 1.885]	0.876

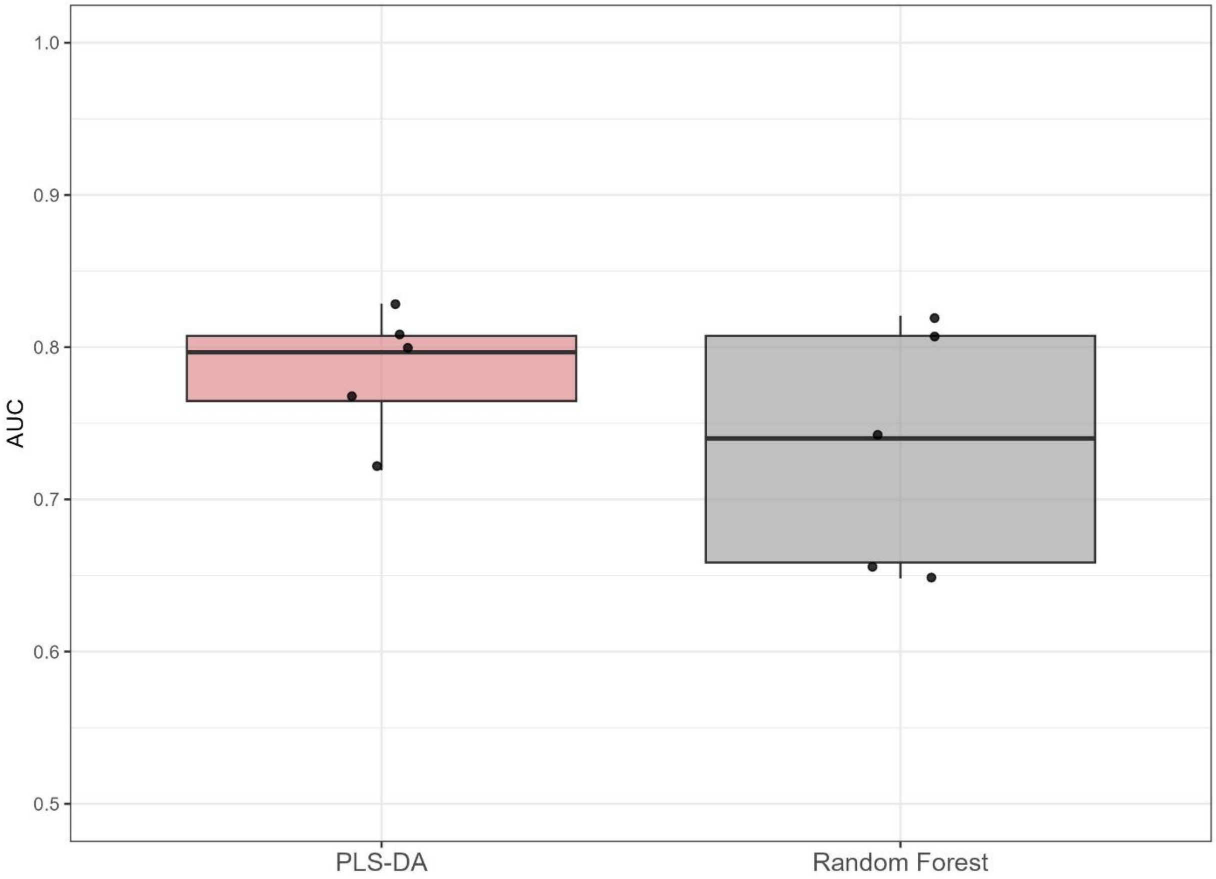

Supplementary Figure 1. **The model performance of PLS-DA and random forest to classify socially deprived frail and non-socially deprived non-frail individuals.** Models were trained on the top 20 proteins identified by SHAP and random forest using 5-fold cross-validation. Boxplot showing the distribution of AUC values across model training folds. Each box represents the interquartile range (IQR), with the horizontal line indicating the median. Individual data points represent the AUC of each fold. The n_component used in the PLS-DA model is n = 2. The optimised parameters for the random forest model after grid search are {n_estimators = 500, max_depth= 20, max_features = 'sqrt', min_samples_split=2}.

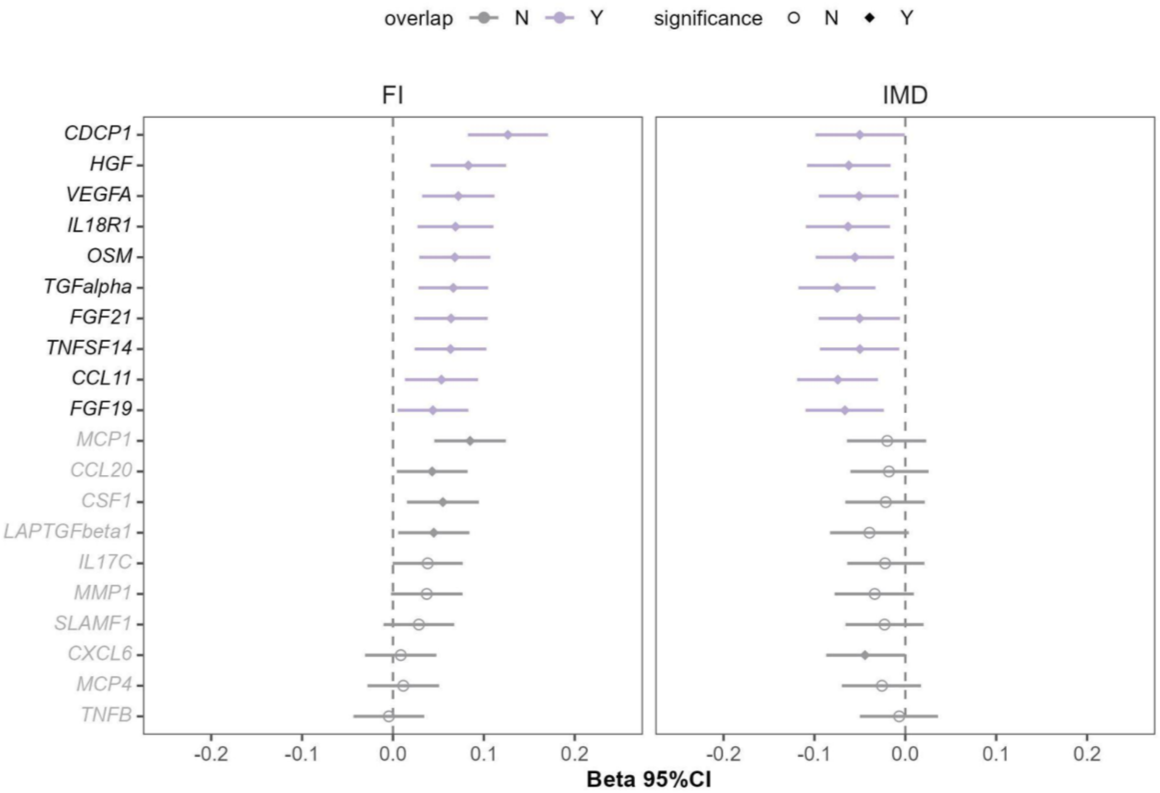

Supplementary Figure 2. The association of inflammation related markers with frailty index and IMD. Beta coefficients were calculated using linear mixed models adjusting for age, BMI, medications for hypertension, diabetes, and hyperlipidaemia, with family relatedness and batch as random effects. Beta [95%CI] is reported for the association of frailty index and IMD with protein signatures in the overall population (n = 2,144). The black font in the y-axis represents the overlapping protein markers for frailty and IMD, and the grey font represents the non-overlapping protein markers.

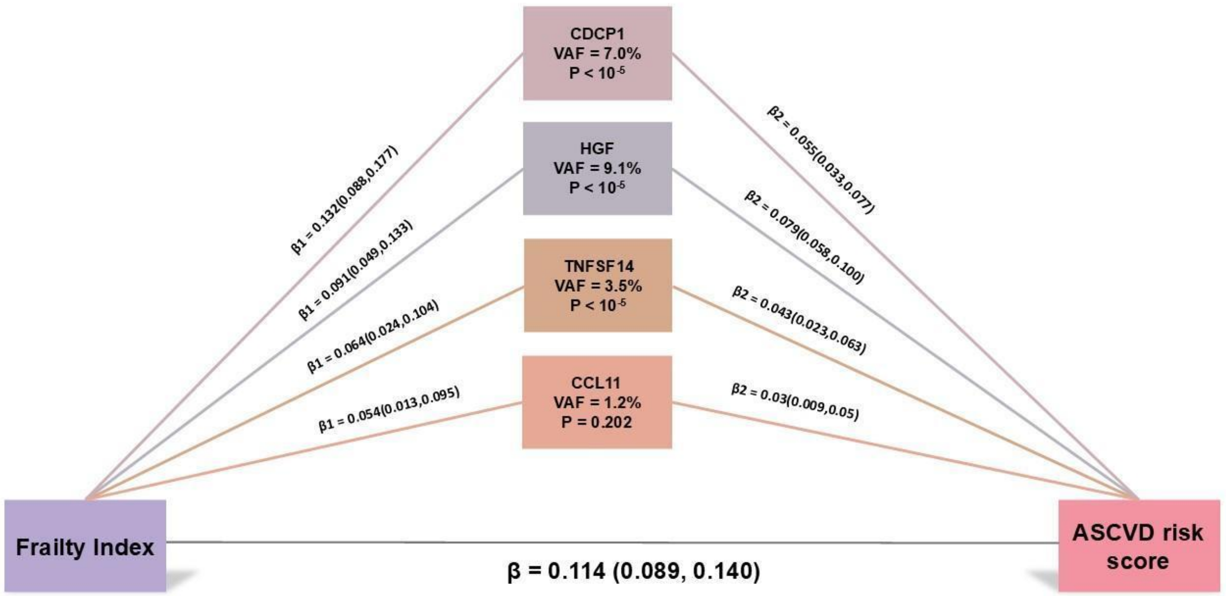

Supplementary Figure 3. Mediation effects of inflammation-related markers on the association between frailty and ASCVD risk adjusting for age and BMI. β1 represents the association between the exposure (frailty index) and inflammatory markers with a 95% CI. β2 represents the association between inflammatory markers and ASCVD score with a 95% CI. β represents the total effect of the exposure (frailty index) on the ASCVD score with a 95% CI. VAF: Variance Account For. P indicates the significance of the indirect effect of mediated protein markers.
