# Supplementary material for: Common inflammatory proteins linking frailty and area-level deprivation as key drivers of cardiovascular risk in women
Source: Commun Med (Lond). 2025 Jul 20;5:301. doi: 10.1038/s43856-025-01012-4 (PMC12276345; doi:10.1038/s43856-025-01012-4)
Supplement: Supplementary file 3 — Description of Additional Supplementary Files [file 43856_2025_1012_MOESM3_ESM.pdf]

Description of Additional Supplementary Files

File name- Supplementary Data 1

File description – Source data for Figure 2B.
