# Supplementary material for: Common inflammatory proteins linking frailty and area-level deprivation as key drivers of cardiovascular risk in women
Source: Commun Med (Lond). 2025 Jul 20;5:301. doi: 10.1038/s43856-025-01012-4 (PMC12276345; doi:10.1038/s43856-025-01012-4)
Supplement: Supplementary file 5 — Reporting Summary [file 43856_2025_1012_MOESM5_ESM.pdf]

nature portfolio

Corresponding author(s): Cristina Menni

Last updated by author(s): Jun 17, 2025



# Reporting Summary

Nature Portfolio wishes to improve the reproducibility of the work that we publish. This form provides structure for consistency and transparency in reporting. For further information on Nature Portfolio policies, see our Editorial Policies and the Editorial Policy Checklist.

## Statistics

For all statistical analyses, confirm that the following items are present in the figure legend, table legend, main text, or Methods section.

| n/a | Confirmed | |
|---|---|---|
| ☐ | ☒ | The exact sample size (*n*) for each experimental group/condition, given as a discrete number and unit of measurement |
| ☐ | ☒ | A statement on whether measurements were taken from distinct samples or whether the same sample was measured repeatedly |
| ☐ | ☒ | The statistical test(s) used AND whether they are one- or two-sided<br>*Only common tests should be described solely by name; describe more complex techniques in the Methods section.* |
| ☐ | ☒ | A description of all covariates tested |
| ☐ | ☒ | A description of any assumptions or corrections, such as tests of normality and adjustment for multiple comparisons |
| ☐ | ☒ | A full description of the statistical parameters including central tendency (e.g. means) or other basic estimates (e.g. regression coefficient) AND variation (e.g. standard deviation) or associated estimates of uncertainty (e.g. confidence intervals) |
| ☐ | ☒ | For null hypothesis testing, the test statistic (e.g. *F*, *t*, *r*) with confidence intervals, effect sizes, degrees of freedom and *P* value noted<br>*Give P values as exact values whenever suitable.* |
| ☒ | ☐ | For Bayesian analysis, information on the choice of priors and Markov chain Monte Carlo settings |
| ☒ | ☐ | For hierarchical and complex designs, identification of the appropriate level for tests and full reporting of outcomes |
| ☐ | ☒ | Estimates of effect sizes (e.g. Cohen's *d*, Pearson's *r*), indicating how they were calculated |

*Our web collection on statistics for biologists contains articles on many of the points above.*

## Software and code

Policy information about availability of computer code

| | |
|---|---|
| Data collection | Data from questionnaires, clinical visits, and laboratory data was entered using comma delimited files, excel spreadsheet and Microsoft access. Proteomic data was measured using the Olink Proximity Extension Assay (PEA) technique as described in the Methods. |
| Data analysis | Statistical analysis was performed using R 4.2.0 and the machine learning models were constructed using Python 3.7.0. The key source codes that supported these results and findings can be found in https://zenodo.org/records/15670583 |

For manuscripts utilizing custom algorithms or software that are central to the research but not yet described in published literature, software must be made available to editors and reviewers. We strongly encourage code deposition in a community repository (e.g. GitHub). See the Nature Portfolio guidelines for submitting code & software for further information.

## Data

Policy information about availability of data

All manuscripts must include a data availability statement. This statement should provide the following information, where applicable:

- Accession codes, unique identifiers, or web links for publicly available datasets
- A description of any restrictions on data availability
- For clinical datasets or third party data, please ensure that the statement adheres to our policy

The data used in this study are held by the Department of Twin Research at King's College London. The data can be released to bona fide researchers using our normal procedures overseen by the Wellcome Trust and its guidelines as part of our core funding (http://twinsuk.ac.uk/resources-for-researchers/access-our-

data/). Data from the Nottingham OA cohort is available upon reasonable request from the principal investigator (ana.valdes@nottingham.ac.uk). Supplementary Data 1 is the source data for Figure 2B. Supplementary Table 2 is the source data for Figure 3A. Supplementary Table 3 is the source data for Figure 3B. Supplementary Table 4 is the source data for Figure 3D.

## Research involving human participants, their data, or biological material

Policy information about studies with human participants or human data. See also policy information about sex, gender (identity/presentation), and sexual orientation and race, ethnicity and racism.

| | |
|---|---|
| Reporting on sex and gender | As indicated in our manuscript, the individuals included in this study are all females, as they were the only group with concurrent measures of frailty, social deprivation level, cardiovascular phenotypes, and proteomic profiling. We have acknowledged in the discussion that the results may not be generalisable to men. |
| Reporting on race, ethnicity, or other socially relevant groupings | All included individuals were of European Ancestry. Info on ethnicity was self reported. The socioeconomic status was measured by Index of Multiple Deprivation(IMD) which is is the official measure of relative deprivation in UK. |
| Population characteristics | Our study included 2,144 females from TwinsUK with concurrent measures of frailty, social deprivation level, cardiovascular phenotypes and proteomic profiling. |
| Recruitment | Study participants were individuals enrolled in the TwinsUK Registry, a national register of adult twins recruited as volunteers without selecting for any particular disease or trait. |
| Ethics oversight | St. Thomas' Hospital Research Ethics Committee |

Note that full information on the approval of the study protocol must also be provided in the manuscript.

# Field-specific reporting

Please select the one below that is the best fit for your research. If you are not sure, read the appropriate sections before making your selection.

☒ Life sciences ☐ Behavioural & social sciences ☐ Ecological, evolutionary & environmental sciences

For a reference copy of the document with all sections, see nature.com/documents/nr-reporting-summary-flat.pdf

# Life sciences study design

All studies must disclose on these points even when the disclosure is negative.

| | |
|---|---|
| Sample size | This study is a posteriori analysis of previously collected data. We included 2,144 individuals with available frailty index, IMD, and proteomic data. A power calculation indicates that with 2,144 individuals, the study has 80% power to detect a correlation coefficient of 0.093 at an alpha level of $5 \times 10^{-4}$. |
| Data exclusions | Individuals without proteomic data available |
| Replication | n = 57 females from the Nottingham cohort |
| Randomization | Not applicable |
| Blinding | Not applicable |

# Reporting for specific materials, systems and methods

We require information from authors about some types of materials, experimental systems and methods used in many studies. Here, indicate whether each material, system or method listed is relevant to your study. If you are not sure if a list item applies to your research, read the appropriate section before selecting a response.

## Materials & experimental systems

| n/a | Involved in the study |
|---|---|
| ☒ | ☐ Antibodies |
| ☒ | ☐ Eukaryotic cell lines |
| ☒ | ☐ Palaeontology and archaeology |
| ☒ | ☐ Animals and other organisms |
| ☒ | ☐ Clinical data |
| ☒ | ☐ Dual use research of concern |
| ☒ | ☐ Plants |

## Methods

| n/a | Involved in the study |
|---|---|
| ☒ | ☐ ChIP-seq |
| ☒ | ☐ Flow cytometry |
| ☒ | ☐ MRI-based neuroimaging |

# Plants

| | |
|---|---|
| Seed stocks | Not applicable |
| Novel plant genotypes | Not applicable |
| Authentication | Not applicable |
